# Cerebrovascular Reactivity: Purpose, Optimizing Methods, and Limitations to Interpretation – A Personal 20-Year Odyssey of (Re)searching

**DOI:** 10.3389/fphys.2021.629651

**Published:** 2021-04-01

**Authors:** Joseph A. Fisher, David J. Mikulis

**Affiliations:** ^1^Department of Physiology, University of Toronto, Toronto, ON, Canada; ^2^Department of Anaesthesia and Pain Management, University Health Network, University of Toronto, Toronto, ON, Canada; ^3^Joint Department of Medical Imaging and the Functional Neuroimaging Lab, University Health Network, Toronto, ON, Canada; ^4^The Joint Department of Medical Imaging, Toronto Western Hospital, University of Toronto, Toronto, ON, Canada; ^5^Techna Institute & Koerner Scientist in MR Imaging, University Health Network, Toronto, ON, Canada

**Keywords:** cerebrovascular reactivity, MRI blood oxygen-level dependent, stroke risk, cerebral steal, hypercapnia, resistance map, speed of response, RespirAct™

## Abstract

The brain is a neurovascular organ. A stimulus-response approach is effective in interrogating the physiology of its vasculature. Ideally, the stimulus is standardized across patients, and in a single patient over time. We developed a standard stimulus and attempted to measure, classify, and interpret the many forms of responses. Over the past 20 years, our work has delivered nuanced insights into normal cerebral vascular physiology, as well as adaptive physiological responses in the presence of disease. The trajectory of our understanding did not follow a logical linear progression; rather, it emerged as a coalescence of new, old, and previously dismissed, ideas that had accumulated over time. In this essay, we review what we believe were our most valuable – and sometimes controversial insights during our two decades-long journey.

## Overview

**Figure 1:**
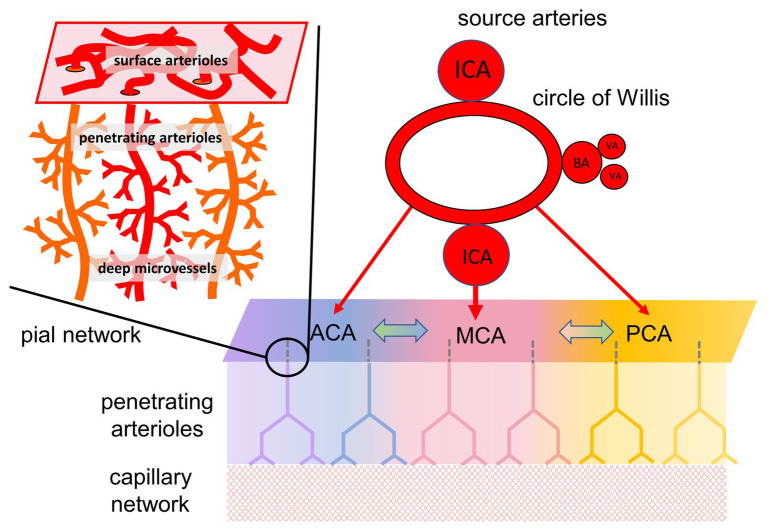
Organization of cerebral blood flow (CBF) and collateral blood flow. CBF is supplied by four extracranial arteries originating from the aortic arch and subclavian arteries, the internal carotid arteries (ICA), and the vertebral arteries (VA). The latter joining to form the basilar artery (BA). Intracranially, these arteries anastomose around the circle of Willis, and separate into the major intracranial vessels perfusing each hemisphere: the anterior cerebral artery (ACA), middle cerebral artery (MCA), and posterior cerebral artery (PCA). Although there are multiple anastomoses between the major cerebral vascular territories, the blood supply to each territory, in baseline states, comes predominantly from its overlying artery (illustrated by color coding). In the case of partial or complete blockage of one of the cerebral arteries, the availability of blood flow depends on the ability to recruit collateral pathways. Figure modified from Fisher et al. (2018).

Brain vascular health relates to a fundamental ability of the cerebrovascular system to match blood flow to tissue demand. This includes providing oxygen and nutrients as well as eliminating metabolic waste. Cerebrovascular physiology has evolved to maintain this supply in the face of reduced perfusion pressure through autoregulation or, when needed, to provide a surge in supply through neurovascular coupling (Willie et al., 2014). The vascular mechanisms affecting these functions are highly coordinated and very effective in health. However, the mechanisms can be compromised under a variety of conditions that harm vessels or reroute blood flow, including steno-occlusive processes, emboli, thrombosis, vascular malformations, tumors, and vasculitis. Under such conditions, adequate perfusion can be restored only when blood flow bypasses the impediment *via* an alternate route, referred to as *collateral blood supply* (Moody et al., 1990; Sobczyk et al., 2020; reviewed in Sheth and Liebeskind, 2015; Figure 1). In the case of upstream obstructions, there is the potential for trophic forces to stimulate the development of new vessels especially in the setting of slowly progressive occlusions as in moyamoya (Scott and Smith, 2009).

The status of collateral blood flow is of particular interest in the presence of known large artery steno-occlusive lesions, especially if accompanied by transient neurological symptoms. Such collateral flow may not be reliably detected by angiography (Ben Hassen et al., 2019). Collateral vessels may not contribute to perfusion if downstream tissue requirements are met *via* the native pathways. Furthermore, the collateral flow passing through tissue arterioles, and capillaries (Moody et al., 1990) is below the resolution of clinical imaging modalities, visible only as a contrast blush on angiography, and exceptionally difficult to quantify. One can, however, follow changes in tissue perfusion through its effect on tissue oxygenation as reflected by the capillary deoxyhemoglobin concentration and its effect on the MRI blood oxygen-level dependent (BOLD) signal.

To test for the presence of *effective* collateral blood flow distal to a steno-occlusive lesion, a vasodilatory challenge must be applied to the system while observing changes in surrogates of blood flow, like the BOLD signal, in the vascular territory of the compromised supply vessel(s). We typically apply an increase in the arterial partial pressure of carbon dioxide (PaCO_2_) to induce global dilation in the cerebral vasculature and observe the patterns of changes in cerebral blood flow (CBF). Assuming tissue metabolic activity remains unchanged (Chen and Pike, 2010a), any changes in BOLD signal reflect predominantly changes in blood flow. The change in BOLD (Δ BOLD), normalized for the change in the stimulus (Δ PaCO_2_), is termed cerebrovascular reactivity (CVR).

Hypercapnia can more than double the resting brain blood flow (Lassen, 1959). Interestingly, the limiting factor in the increase in CBF is the inflow capacity of the extracranial carotid and vertebral arteries (Faraci and Heistad, 1990), in effect setting dilating downstream intracranial vessels in competition for the inflow. In health, hypercapnia-induced regional reductions in resistance are balanced among vessels and vascular territories, resulting in an orderly distribution of blood flow throughout the brain (Bhogal et al., 2014). But in the presence of localized hemodynamic disturbance, such as with a steno-occlusive lesion, localized vasculitis, and other structural or metabolic disorders, global cerebral vasostimulation exposes and amplifies the imbalances in flow distribution. The patterns of flow distribution reflect the summed effects of (i) the strength of the stimulus, (ii) the upstream hemodynamic impediment, (iii) the residual local (downstream) vasodilatory reserves, and (iv) the downstream vasculature’s access to collateral flow pathways. When we began these studies at the turn of the millennium, we could not sort out these effectors, and we were left to observe a variety disrupted flow patterns in patients with cerebrovascular impairment. We nevertheless remained determined to assemble such observations and over time, conceptually order them, and reorder them, into a chain of adaptive processes that reveal new insights into neurovascular physiology, and explain the pathophysiology of disease. In the following section, we will review some of the highlights of this journey.

## Cerebrovascular Reactivities

### Basic Cerebrovascular Reactivity

On the most basic level, CVR performed with MRI follows a voxel-wise Δ BOLD normalized to the vasoactive stimulus, Δ PaCO_2_. The limitations of Δ BOLD, as a surrogate of Δ flow have been discussed at length elsewhere (Hoge et al., 1999; Chiarelli et al., 2007; Fierstra et al., 2018). We, like many others, initially made the simplifying assumption that Δ BOLD reflects the change in flow in vessels within each voxel and, therefore, the underlying change in their vascular diameter. We ignored changes in perfusion pressure which, in any event, we could not measure. In the work of other contemporary laboratories, CVR was reported as only the amplitude of response to a stimulus, Δ BOLD, while the magnitude of the stimulus, Δ PaCO_2_, was unknown, e.g., with breath-holding, and carbogen administration (Mark et al., 2010; Fisher, 2016), or resulting from intravenously administered acetazolamide (reviewed in Fierstra et al., 2013).

We began our studies around 1997 by studying patients with cerebrovascular steno-occlusive disease. The sought-after stimulus at that time was square wave “boxcar” changes in end-tidal PCO_2_ (PETCO_2_) to minimize the effect of the known drift of baseline BOLD signal (Vesely et al., 2001), a solution used in functional MRI. Our focus then was to use the reductions in BOLD signal in the territory distal to the vascular occlusion as a biomarker of the severity of hemodynamic compromise. The reduction in flow was referred to as “vascular steal” (Brawley, 1968; Symon, 1968) analogous to “subclavian steal” (Reivich et al., 1961). Over the next decades, steal was considered a sign of increased vulnerability to ischemic stroke (Markus and Cullinane, 2001; Reinhard et al., 2008; Gupta et al., 2012) with the potential to inform decisions about patient management. During this time, we sorted through a number of confounders. The puzzling “steal” seen over the ventricles was eventually explained by Thomas et al. (2013). We also found that various aspects of the stimulus influenced the flow pattern: size of the stimulus (Δ PETCO_2_), baseline PETCO_2_, direction of PETCO_2_ change, rate of change of PETCO_2_, and others (Sobczyk et al., 2014).

**Figure 2:**
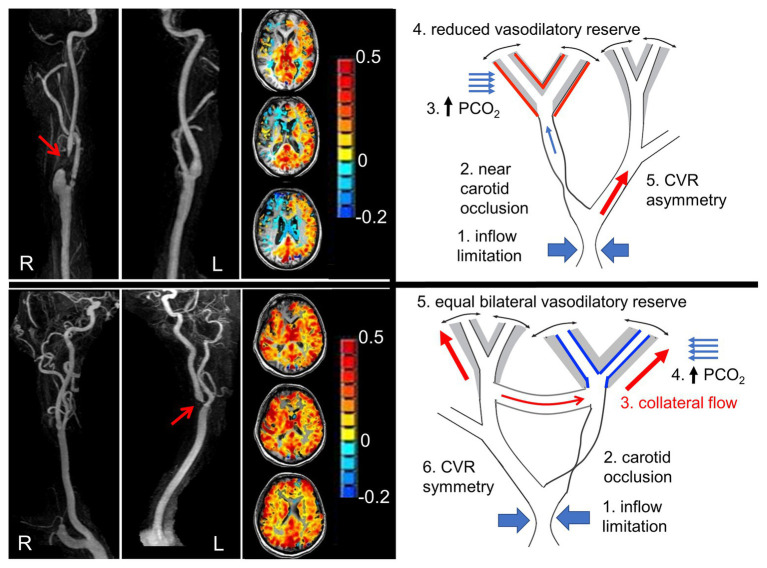
Pathophysiology of steal, and steal as a marker of availability of collateral flow. **Left panel** shows angiograms from two patients with nearly occluded carotid arteries (red arrows). Next to each are axial slice cerebrovascular reactivity (CVR) maps from the hypercapnic test. The CVR for each voxel is color coded according to the color scheme shown and mapped onto the corresponding voxel of the anatomical scan. The **upper panel** shows steal physiology (blue voxels on axial scan), and the **lower panel** shows near normal CVR. The suggested mechanism of each map is shown in the schematic to the right. **Upper right panel**: (1) Flow capacity of intracranial vessels greater than can be supplied by extracranial vessels depicted as “inflow limitation”; (2) occlusion of a branch; (3) hypercapnia and stimulation of vasodilation; (4) vasodilatory reserve (gray area) is exhausted downstream from the occlusive lesion (indicated by the red lines on the outside of the gray zone), but intact on the contralateral side (indicated by black lines inside gray zone); and (5) with hypercapnia, flow is directed predominantly to the contralateral side, dropping the perfusion pressure at the bifurcation. The reduced perfusion pressure reduces the flow downstream from the lesion. In the **lower right panel**: (1) Collateral flow bypasses the stenotic lesion. Both sides retain flow reserve. A hypercapnic stimulus results in bilateral positive CVR. Modified from Hoiland et al. (2019).

From the time of our earliest investigations, we were troubled that the extent and severity of steal did not always match the degree of stenosis in large vessels. Indeed, we were puzzled by patients with totally occlusive vascular lesions on angiography, but little or no steal, and few or no symptoms. Our working hypothesis was that, in these patients, the blood was bypassing the stenosis and arriving at the parenchyma *via* another route, i.e., collateral flow (McVerry et al., 2012; Alves et al., 2016; Fisher et al., 2018; Figure 2). This hypothesis was investigated in our laboratory by Sobczyk in a series of studies that were part of her doctoral dissertation.^2^ She has recently published additional supportive data that challenges the traditional role of the measurement of residual lumen diameter as the primary determinant of stroke risk (Sobczyk et al., 2020). The main implication of this collective work is that the presence of *effective* collateral flow, rather than the degree of carotid artery narrowing, explains why CVR more accurately predicts the risk of stroke than degree of stenosis alone (Gupta et al., 2012; Reinhard et al., 2014).

### Advanced Cerebrovascular Reactivity: The Clues to Underlying Physiology

The “box car” stimulus approach was initially very helpful in the analysis of the data, using a “steal/no steal” paradigm to reduce system complexity into a binary assessment with implications for severity of underlying pathology, risk of stroke, and advisability of surgical revascularization. Throughout this period, we also tried to address other aspects of the complexity of CVR by re-examining our initial assumption that Δ BOLD was sufficiently normalized and standardized if simply divided by Δ PETCO_2_. For further discussion on optimizing the stimulus for interpretation of physiology.

#### Speed of Response

The ability to generate, literally, square wave changes in PETCO_2_ gave us many opportunities to observe the delays between the change in PETCO_2_ and that of raw BOLD signal. We were soon able to predict the processed CVR maps from these data noting that the BOLD signal of healthy vasculature follows PETCO_2_ very closely in time and amplitude, but the BOLD response of compromised vasculature is delayed and dampened. The standing model to explain lags in vascular flow response to changes in PaCO_2_ is that of Thomas et al. (2014). In this model, smooth muscle tone responds to the tissue PCO_2_ which is a function of the rate of buildup and elimination of carbon dioxide (CO_2_) in the interstitial and intracellular fluid surrounding the vessel. These extravascular tissues are considered to be a CO_2_ reservoir which has a CO_2_ capacitance that buffers the amplitude and rates of change of vascular CO_2_ concentrations and thereby, blood flow. With the publication of Poublanc et al. (2015), we introduced a model whereby vascular smooth muscle responds directly to intra-arterial hydrogen ion concentrations ([H+]). The model holds that with hypercapnia, intra-arterial H+ diffuses into the vascular smooth muscle cells, reduces intra-cellular [Ca^++^] (Swietach et al., 2013) and relaxes smooth muscle tone (Peng et al., 1998; Boedtkjer, 2018), thereby increasing blood flow. We modeled the flow response to CO_2_ as a first order exponential where following an abrupt change in arterial [H+], the *τ* of the BOLD response as it approaches a new equilibrium level represents the fitness of the smooth muscle tone control, with longer *τ* representing greater dysfunction (see Duffin et al., 2021).

The simplifying assumption of the vascular response being of a first order exponential led to our convolving the actual PETCO2 changes over the duration of the stimulus with (*τ*) ranging from 2 to 100 s. The speed of response was designated by the exponential that provided the best fit when regressed against the actual BOLD signal. Tau provided a complementary metric to the steady state CVR which is just a measure of the magnitude of response. Duffin from our laboratory showed the phase response from transfer function analysis (TFA) provided a similar measure (Duffin et al., 2015). Indeed, TFA, in addition to *τ*, has become a standard feature of our post-processing. Dynamic responsiveness has proven to be informative in the assessment of steno-occlusive disease (Poublanc et al., 2015), amnestic mild cognitive impairment and Alzheimer’s disease (Holmes et al., 2020), concussion (Shafi et al., 2020), and sickle cell disease (Forte et al., 2020). We currently believe that the speed of vascular response indicated by *tau* is a critical element of healthy vascular performance. Tau will also emerge as a highly sensitive biomarker for the severity of diseases that disrupt vessel structure and degrade vascular performance such as diabetes, hypertension, hyperlipidemia, smoking, i.e., those that are associated with hyalin sclerosis in arterioles.

Dynamic responsiveness (i.e., *τ*) cannot be assessed using such vasoactive stimuli as acetazolamide injection, breath hold, or fixed inspired CO_2_ (e.g., carbogen). The time courses of change in stimulation for acetazolamide, and the rise of CO_2_ with these methods, are considerably longer than those of the vascular responses, substantially obscuring them. Moreover, with these methods, PETCO_2_ is not equal to the PaCO_2_ (Jones and Jurkowski, 1979; Robbins et al., 1990), the actual independent variable, so the stimulus is not precisely known.

#### Voxel Flow Measurement and Voxel Vessel Diameters

##### What Flow Surrogate Measure to Use?

A suitable flow measure for CVR would require high temporal and spatial resolution. Ultrasound Doppler has the former, but not the latter. Furthermore, the velocity measure does not reliably reflect flow because of large changes in diameter of extracranial and intracranial arteries with changes in PaCO_2_ (Al-Khazraji et al., 2019).

MRI arterial spin labeling (ASL) using a flow-sensitive alternating inversion recovery (FAIR) sequence has 4 s temporal resolution and has been well correlated with positron emission tomography (Chen et al., 2008). It also works well in patients with vascular disease (Mandell et al., 2008). However, FAIR’s truncated spatial resolution to a few slices makes it problematic when whole brain resolution is required. ASL is also confounded by tagged blood arrival delays in patients with dyssynchronous intracranial blood arrival times that occur with cerebrovascular disease. Synchronously tagged blood delayed in collateral paths may arrive in the microcirculation after considerable loss of the spin tag due to T1 relaxation effects, significantly reducing signal to noise ratio. Active research in this area is expected to provide solutions and when they become available, ASL will be a preferred option for measuring CVR.

The BOLD signal is an acknowledged imperfect surrogate for flow. First, the relationship of BOLD to flow is not linear but exponential, rising with increasing flow only at the rate of progressive dilution of a constant amount of deoxyhemoglobin formation (Chiarelli et al., 2007). In addition, BOLD is affected by changes in cerebral blood volume (controlled for by Grubb’s constant; Grubb et al., 1974; Chen and Pike, 2010b) and any changes in oxygen consumption (Chen and Pike, 2010a). Nevertheless, we settled on BOLD MRI for measuring flow changes. It has the advantage of high temporal resolution (1 s), whole brain coverage, and a reasonably linear response in the range of CO_2_ stimuli applied for measuring CVR metrics (Hoge et al., 1999).

##### Should All Voxels Exhibit Sigmoid Flow Responses?

In time, we had to reconsider our first, seemingly necessary, simplifying assumption that measured *changes in voxel flow reflect changes in the diameters of the vessels of the corresponding voxel*. This assumption at first blush seems fundamentally reasonable. But, we nevertheless had doubts, even as we adopted it. We put these doubts aside, but not out of mind. We reconsidered the issue repeatedly over the years. First, we laid out fundamental concepts concerning blood flow: changes in blood vessel diameter have a limited range. At the extremes of the range, the change in diameter per unit change in stimulus is reduced, resulting in a sigmoidal relationship between flow and PaCO_2_ for a given perfusion pressure. Indeed, the flow response to PaCO_2_ is sigmoidal in healthy humans (Bhogal et al., 2014, 2015, 2016; Duffin et al., 2019).

**Figure 3:**
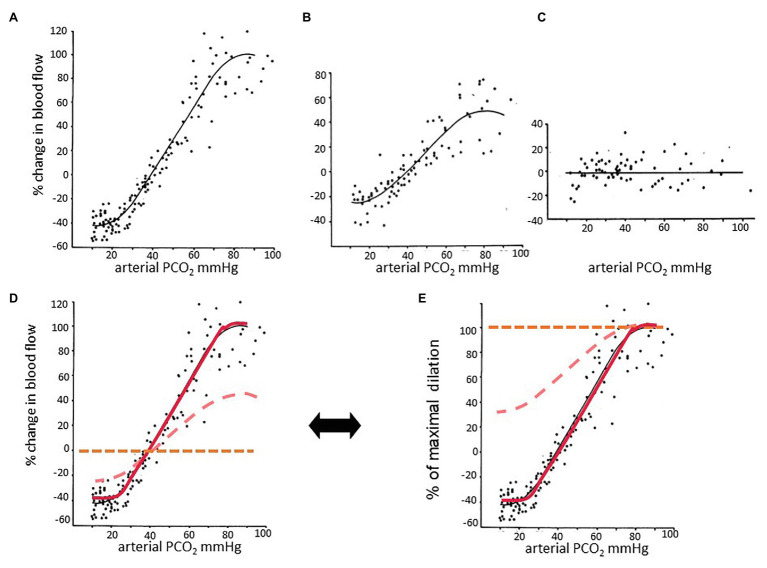
The effect of partial pressure of carbon dioxide (PaCO_2_) on blood flow at different perfusion pressures. Data taken from Harper and Glass (1965). In this experiment, the authors anesthetized dogs and measured CBF by washout of radioactive counts. Graphs in the top row were taken from the paper and adjusted to the same scale. **(A)** Normotensive; **(B)** mild hypotension; **(C)** severe hypotension. **(D)** The three graphs are plotted on the same axes. **(A)** The relationship between PaCO_2_ and CBF is sigmoidal. **(B)** The encroachment on the vasodilatory reserve by perfusion pressure autoregulatory-induced vasodilation reduces the reactivity to carbon dioxide (CO_2_), as indicated by the slopes of the linear part of the sigmoid curves. Perfusion pressure autoregulation and CO_2_ reactivity compete to alter vessel diameter. **(C)** When autoregulation is exhausted, there is no further response to hypercapnia and little or no constriction to hypocapnia. **(D)** The midpoints of the sigmoidal curves are approximately at the resting PCO_2_. **(E)** Percent of maximal dilation. At high PCO_2_, or low blood pressure, vessels are maximally dilated. Healthy vessels in normotensive subjects maintain vascular tone with considerable vasodilatory reserve. Vessels maximizing their autoregulation have minimal vasodilatory reserve. Thus a hypercapnic stimulus of sufficient magnitude will continue to dilate healthy vessels while those that have exhausted their vasodilatory reserve will increase their flow to a lesser extent, or even reduce their flow and suffer steal.

**Figure 4:**
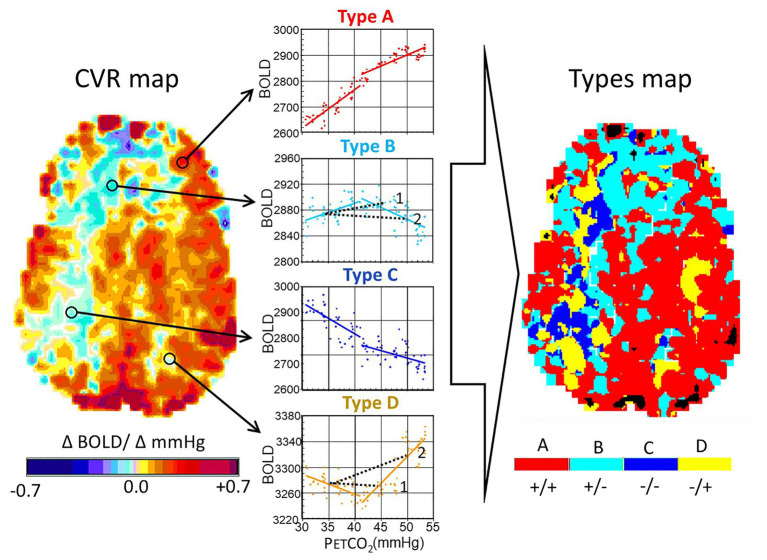
Analysis of ramp hypercapnia data. Stimulus paradigms in an 18-year-old male subject with a history of moyamoya disease affecting the right middle cerebral artery and both anterior cerebral artery territories. The end-tidal PCO_2_ (PETCO_2_) was initiated at baseline PETCO_2_, reduced to 30 mmHg by asking the subject to hyperventilate, and then raised at a constant rate, regardless of minute ventilation or breathing pattern, resulting in a linear rise of PETCO_2_ vs. time, to 55 mmHg over 4 min. We examined the blood oxygen-level dependent (BOLD) signal as a function of PCO_2_. The interrogated voxel locations are indicated by the circles on the CVR maps. Types of response: A, positive response with a sigmoidal shape; B, initial positive response which then declines; C, response which progressively declines; and D, response that initially declines but then increases. In healthy people, A Type voxels overwhelmingly predominate, and the map is substantially red. In this patient, voxels are color coded according to the color of the graph corresponding to the Type. Note the projection of CVR as would have been calculated for a 2-point PETCO_2_ stimulus of 45 and 50 mmHg for the voxels of Type B and D (see 1 and 2). In the upper figure, a PETCO_2_ stimulus of 45 mmHg results in a positive CVR but at a PETCO_2_ of 50 mmHg, CVR of the same voxel is negative, indicating “steal.” In the lower figure, PETCO_2_ of 45 mmHg results in a negative CVR and at 50 mmHg, it is positive. Modified from Duffin et al. (2017).

Based on the literature, we expected the same response in the presence of vascular disease. Earlier animal studies by Harper and Glass (1965) and later human studies by Ringelstein et al. (1988) used the flow in the whole brain as a model of blood flow response to PaCO_2_ (Figure 3 and also Sobczyk et al., 2014) at various degrees of reduced perfusion pressure. In those studies, reductions in perfusion pressure flattened the sigmoidal flow-response curves. We generalized these observations in considering that the variations in amplitude and other characteristics of a sigmoidal curve (Figure 4) reflect the integrity of a vessel’s anatomy and physiology. As the vessel’s ability to respond to the PaCO_2_ wanes, the sigmoid response curve flattens: both the amplitude and slope of the sigmoid decrease. We hypothesized that this would also apply at the level of a local group of vessels represented in an imaging voxel.

##### Seems That Some Voxels Do Not Exhibit Sigmoid Flow Profiles

We tested this intuitively satisfying hypothesis by applying a ramp increase in PaCO_2_ in patients with cerebrovascular pathology, where the ramping of PaCO_2_ is intended to capture most of the full sigmoidal range of response. Some of the patients with steno-occlusive disease indeed showed regional steal. When we checked the shape of the responses at the level of the voxel, many voxels in the non-steal territories had sigmoidal responses, but many others had responses that were clearly not sigmoidal – rather they were bimodal, particularly in areas with poor CVR and steal. In these regions, voxels could be classified into one of four general patterns (Figure 4). This surprising finding challenged our previous assumptions that the slope of a straight line fit to two points of the PaCO_2_-BOLD relationship was a suitable interpretation of the vascular response (Figure 4, dotted lines in Type B and Type D). The abundance of these four types of patterns initially challenged what we ultimately came to accept as axiomatic: *the vascular resistance response of all vessels to progressive hypercapnia is indeed sigmoidal, but the flow, or BOLD signal, changes may not be!*

##### A Change in Concept From Sigmoidal *Flow*, to Sigmoidal *Resistance*

To reconcile the observations of types of responses with the understanding of the foundation of vascular physiology, we had to jettison the belief that the measured changes in flow reflected only changes in vascular diameter in the *underlying* voxels. The transformative insight for us was that in patients with cerebrovascular disease, the observed pattern of the changes in flow to a progressively increasing stimulus resulted from the combination of (i) local changes in vascular diameter, as determined by its vasodilatory reserve which is sigmoidal in shape (Sobczyk et al., 2014), and (ii) the uneven distribution of perfusion pressure.

**Figure 5:**
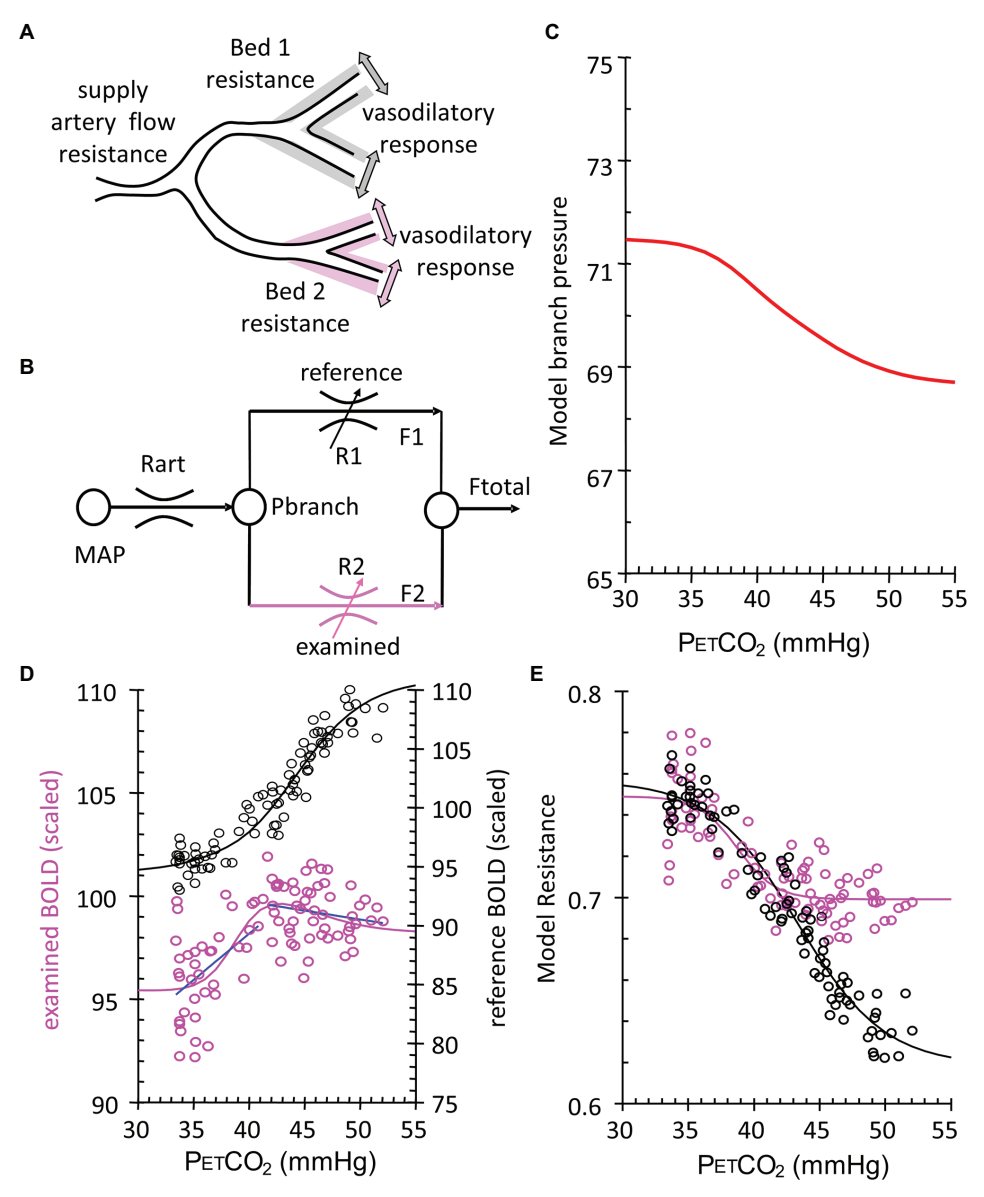
Calculation of voxel resistance sigmoidal profile over range of PETCO_2_. **(A)** Theoretical illustration of two brain vascular territories both supplied in parallel by a major artery with resistance (flow limitation). Their vasodilatory responses are shown as shading. **(B)** Simple resistance circuit model. Reference and examined vascular beds with resistances R1 and R2 are perfused *via* an arterial flow resistance (Rart) from mean arterial blood pressure (MAP). The pressure perfusing the two branches (Pbranch) and their respective resistances establishes flows through each branch (F1 and F2) that sum to Ftotal. Given F1 and F2 as BOLD measures for the reference and examined voxels, R1 and R2 can be calculated by considering the Model as an analog of an electrical circuit. The Model assumes that this unit may be expanded and contracted in a fractal pattern to contain the blood supply of both hemispheres, from extracranial vessels to capillaries. Every vessel or vascular bed is considered in fluid continuity with every other vessel. As such, we use the examined bed for each unit on a voxel scale, as if it is perfused in parallel with an ideal voxel of full vasodilatory responsiveness. Pbranch values result from the assumption of MAP = 100 mmHg and the pressure loss in Rart assumed to be 30 mmHg (Faraci and Heistad, 1990). Regardless of flow pattern Type (Figure 4), the calculation invariably results in a sigmoidal resistance response to PETCO_2_. **(C)** Model branch pressure change with PETCO_2_. **(D)** Measured flow responses to PETCO_2_ (purple open circles and blue Type line fits) and the reference voxel (black open circles). **(E)** Model resistance-CO_2_ responses calculated from the Model flow responses and the reference voxel flow. Sigmoid curves (lines) are fitted to the resistance-CO_2_ responses for each voxel.

Had the cerebral inflow potential from the extracranial vessels not been limited (as in the aforementioned studies of Harper and Glass, 1965; Ringelstein et al., 1988), possibly all observed flows would indeed reflect the changing vascular diameters and consequent flow resistance of the voxel’s underlying capillaries (Figure 2). But in the presence of restricted inflow and stable systemic blood pressure, local perfusion pressures and thus brain flow distributions become dependent on the pattern of regional flow resistances in the way an electrical current would be by wiring together multiple electrical resistances in parallel, with CVR and steal being a hydraulic analogy of Ohm’s law for electrical resistances (Figure 5). As the vasodilatory stimulus increases, the blood flow will shift to the vessels that can continue to lower their resistance.

**Figure 6:**
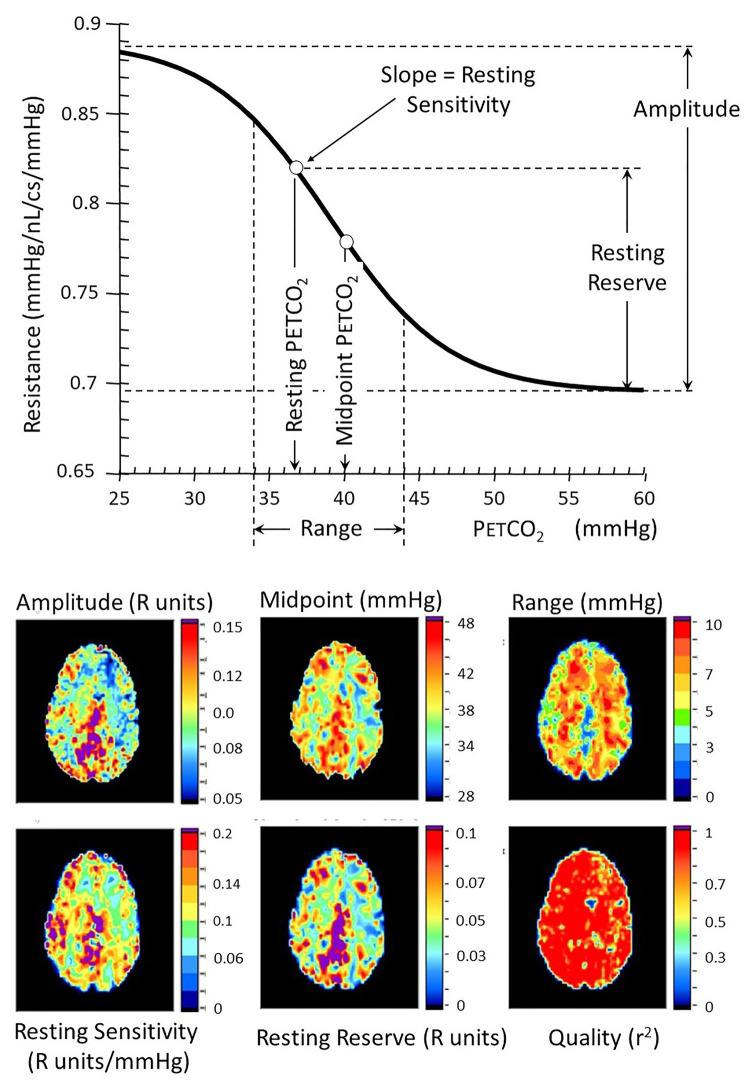
Resistance sigmoid measures **(top)** and their corresponding anatomical maps **(bottom)**. The vascular model is that described in Figure 5. Various aspects of the resistance-PETCO_2_ relationship are expressed in the sigmoidal shape of the relationship. The graph shows some of these parameters diagrammatically, and the maps show their anatomical distribution. The amplitude shows the full extent of the vasodilation and vasoconstriction. The midpoint of the sigmoid is the PETCO_2_ at its steepest slope, and the range is the span of PETCO_2_ over which the response can be considered linear. The reserve and sensitivity show the vasodilatory reserve available from the subject’s resting baseline PETCO_2_ and the response sensitivity (sigmoid slope) at resting PETCO_2_. The quality map shows the *r*^2^ fit of the examined voxel resistance sigmoid.

We followed up on this insight by modeling regional CBF as a fractal assembly of simple units of two flow resistances in parallel (Figure 5B). The Model, meticulously assembled by Duffin, uses the data of BOLD vs. PETCO_2_ that includes the two biphasic flows to back-calculate the pattern of vascular resistance in the underlying voxel over the range of the ramp stimulus (Duffin et al., 2017, 2018; Fisher et al., 2017). We found that the PETCO_2_ vs. resistance curve is indeed sigmoidal in shape, regardless of the net flow pattern, or more specifically flow Type, and vice versa! The sigmoidal shape of the resistance, and not the blood flow changes themselves, is, therefore, a fundamental property of the vasculature. As such, the descriptions of the vascular physiology lend themselves to be summarized by terms describing the mathematical properties of the sigmoid curves (Figure 6).

To summarize this section, we had progressed from assuming that the BOLD change reflects the underlying voxel’s linear change in resistance to calculating its actual resistance profile by accounting for the net inflow and the net resistance changes of the vessel’s connected network. In doing so, we find all resistance profile calculations yield sigmoidal relationships, regardless of the observed pattern of flow response to progressive PETCO_2_. This provides confidence that our calculated correction reflects the underlying vascular physiology.

## Requirements For the Clinical Application of CVR

Many forms of CVR have been described using a variety of stimuli and flow assays. All such studies published to date have used these CVR measures to compare cohorts of subjects. Broad questions can be addressed by examining well-matched groups to cancel the “noise” of randomly distributed variables and shortcomings of the test. Despite its merits, this approach has low sensitivity in discerning an abnormality in an *individual* patient.

To be suitable for “clinical assessment” implies that a CVR test in a single individual could be interpreted as “normal” or “abnormal.” For this, one needs criteria and thresholds for normality and abnormality as in, for example, blood clotting tests. One needs to be able to address questions such as: (1) “Is the CVR test of Mr. Jones within the normal range?,” (2) “Does his CVR suggest that he would benefit from cerebral bypass surgery?,” (3) “How certain can we be that his surgery improved his vascular status as measured by CVR?” These questions have not yet been addressed due to the inherent imprecision of the stimuli used in most studies of CVR: breath holding, fixed inspired PCO_2_, or acetazolamide.

**Figure 7:**
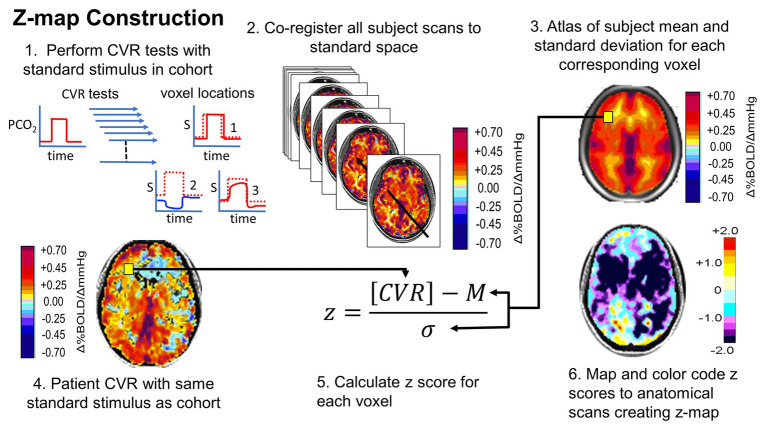
Normalizing the CVR score in terms of the normal range. An identical, repeatable stimulus is applied to a healthy cohort. CVR is calculated and mapped to standard space. Mean (M) and SD are calculated for each voxel. A patient is administered the same test. The patient CVR is assessed as *z*-scores. *Z*-scores can be color coded and mapped over the anatomical scan to show the distribution of abnormality (reprinted from Fisher et al., 2018 with permission of the publisher).

A requirement for any clinical assessment is a standardized repeatable test that includes the same baseline, the same level of change in PaCO_2_ from baseline, the same pattern of change over time, and the same duration applied to repeated patient examinations. This standardized test can then be applied to a representative population and examined in standard anatomical space to define the “normal” population, its mean value (*m*) and its variance, the *SD*. A patient’s CVR can then be scored using the *z*-score as the difference between their result and the population *m*, divided by the *SD*, giving the probability that the test value falls in the normal range (Sobczyk et al., 2015). This process, also developed by Sobczyk during her doctoral thesis work, is summarized in Figure 7. The analogous process can be applied to follow CVR in a single patient over time, to monitor either the natural history of the disease or the effects of treatment (Sobczyk et al., 2016).

### Reproducibility in CVR Testing

#### Reproducible Stimulus

Administering a reproducible stimulus is easy. Obtaining a reproducible physiological effect is very difficult.

##### Acetazolamide

Acetazolamide (ACZ) infused intravenously, blocks the conversion of carbonic acid (H_2_CO_3_) to CO_2_ and H_2_O leaving brain tissue somewhat acidified and resulting in vasodilation. ACZ has been administered in supramaximal doses (1.5 mg/kg) with the expectation of a uniform maximal hyperemic response across subjects. Unfortunately, there is uncontrolled individual variation in the magnitude of response, its timing, and ability to maintain isocapnia as the CBF continues to respond to changes in PaCO_2_. The slow onset time also precludes assessment of the speed of the vascular response to a stimulus. At best, it can be characterized as a binary “on/off” stimulus (albeit without certainty as to timing and maximal effect) so that, as defined, it has significant limitations for single subject clinical application.

##### Carbon Dioxide

Carbon dioxide is readily available in gaseous form, is safe, inexpensive, and in the absence of hypoxia, has no life-threatening side effects at up to more than triple the baseline PaCO_2_ (Feihl and Perret, 1994).

###### Fixed Inspired CO_2_ Concentration.

PaCO_2_ is determined by both the fraction of CO_2_ in inspired gas (FICO_2_) and the subject’s ventilatory response, termed minute ventilation (⩒E). The former is under the control of the investigator; the latter, however, is uncontrolled, unpredictable, and mostly unmeasured, resulting in an uncontrolled and unpredictable PaCO_2_ (Baddeley et al., 2000; Peebles et al., 2007; Fisher, 2016).

###### Precise Targeting of PaCO_2_.

Clinical, individual testing requires the ability to accurately implement a target PaCO_2_, the true independent variable, and to do so independently of the major confounder, ⩒E.

i. Dynamic end tidal forcing

In 1982, Robbins (Robbins et al., 1982) described a breath-by-breath feedback method to target PETCO_2_ by controlling the inspired concentration of CO_2_ according to the previous PETCO_2_. An MRI-compatible system was later described (Wise et al., 2007). However, this system is cumbersome, complex, wasteful of source gases, expensive, and not available in assembled form and so must be custom built to be adopted. The relation between the PETCO_2_ and the PaCO_2_ remains unverified (Jones et al., 1979; Robbins et al., 1990).

ii. Sequential Gas Delivery (1997+)

**Figure 8:**
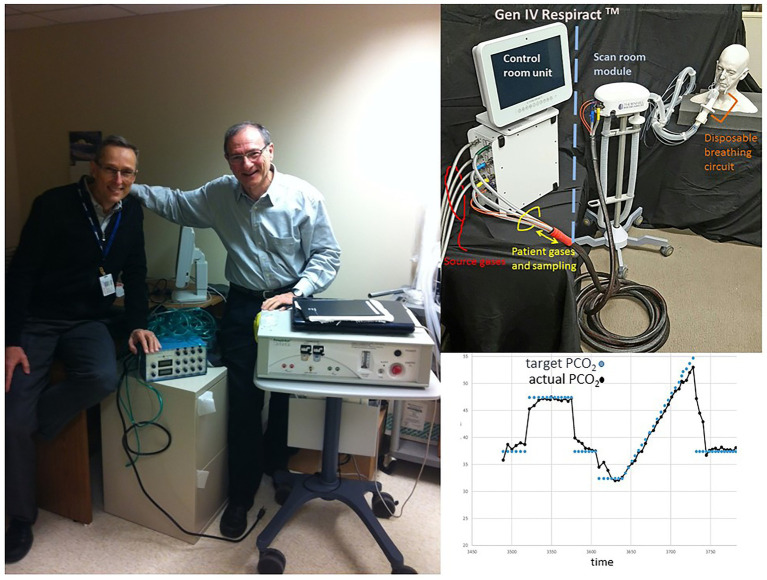
The authors and the generations of gas control devices. **Left**: DM with hand on Generation 1 end-tidal gas control device used in 1998. JF with hand on Generation 3 gas control device, by then called, RespirAct™. **Top right**: Generation 4 RespirAct™. **Bottom right**: Step-ramp paradigm implemented with Generation 4 RespirAct™.

At the end of the last century, one of us (JF) was developing a technique of sequential gas delivery (SGD) which enables the delivery of a precise volume of gas to the gas exchange part of the lung regardless of breath size or breathing pattern (Sommer et al., 1998), and was searching for clinical applications. At the same time, DM, a new staff neuroradiologist at the same university (Figure 8), was performing BOLD fMRI studies that used square-wave changes in neuronal activation for augmenting blood flow detectable with BOLD MRI for assessing neurovascular coupling to detect neural networks. He realized that the ability to generate a quantitative repeatable square-wave vasodilatory stimulus using CO_2_ would have considerable potential for interrogating the entire cerebrovascular system in individual patients with cerebrovascular disease. He was seeking an MRI compatible method of precise gas control for the development of MRI based CVR as a clinical tool and was referred to JF by a colleague.

Prospectively targeting PaCO_2_ is straight forward in theory. The approximate lung volume [functional residual capacity (FRC)] is known from nomograms that take as inputs age, height, weight, and sex. PCO_2_ in the FRC can be measured as it is equal to PCO_2_ in exhaled gas. CO_2_ production from metabolic activity can also be measured (the sum of exhaled PCO_2_ integrated for flow/time). It is then straightforward to calculate the volume and concentration of CO_2_ needed to be inhaled in order to attain any target PCO_2_ in the lung at the end of inspiration (Slessarev et al., 2007).

However, it is not straightforward to restrict inspiration to a discreet volume of CO_2_-containing gas such that this discrete volume, and only this volume, is distributed to the alveoli, and only to the alveoli (excluding the airways). The early evolution and proof of concept of these methods from basic principles over a decade was amply abetted by a large host of colleagues and graduate students, all co-authors of the early publications on the topic (e.g., Sommer et al., 1998; Vesely et al., 2001; Somogyi et al., 2005; Slessarev et al., 2007). A method of administering such a volume has an additional interesting property: It is the only method in which the PETCO_2_ is equal to PaCO_2_, within measurement error, over a large range of PETCO_2_ in healthy spontaneously breathing humans (Ito et al., 2008) and in newborn and adult ventilated animals with severe lung disease (Fierstra et al., 2011, 2012). This unprecedented precision and accuracy of targeting blood gases enabled the reproducibility, and hence the standardization of the testing, which led to the establishment of normal ranges for CVR voxel by voxel (Sobczyk et al., 2015, 2016), normal test-test variability (Sobczyk et al., 2016), and increased sensitivity enabling, in addition to steno-occlusive disease, the study of the microvascular disease in white matter (Sam et al., 2016a,b,c), dementia (Holmes et al., 2020), and vascular dysfunction of traumatic brain injury (Mutch et al., 2015; Ellis et al., 2016; Shafi et al., 2020).

Surprisingly, despite the hard work, insights, and calculations, the crucial components for successful prospective targeting of PaCO_2_ with SGD are very simple, inexpensive, and mundane, and, in retrospect, could have been easily accomplished more than 75 years earlier (Fisher et al., 2016). The bigger wonder is that considering their well-described limitations with respect to data acquisition, results, and interpretation, some investigators prefer using breath holding, carbogen, and ACZ as provocative stimuli.

## Summary

The patterns of blood flow distribution in the brain in response to a vasoactive stimulus can be complex in the presence of cerebrovascular disease, but they are nevertheless the only comprehensive output data we can access. Any measures we make, interpretations we proffer, and conclusions we come to, can emanate only from analyzing these data. At the most fundamental level, we seek to distinguish “abnormal” from “normal” patterns of flow. To accomplish this aim, we identified a single repeatable vasoactive stimulus and a surrogate measure of CBF. We then performed CVR tests on a healthy cohort to establish normal values. This enables the identification and scoring of pathology in individual patients. Ultimately, we applied these data to understand how the observed patterns of flow revealed vascular pathophysiology in terms of perfusion pressures and vascular diameter summarized as vascular resistance. This essay is an overview of our progress in that regard, over the past two decades.

## Data Availability Statement

The raw data supporting the conclusions of this article will be made available by the authors, without undue reservation.

## Ethics Statement

All data involving human participants were reviewed and approved by Ethics Review Board University Health Network, Toronto. All patients/participants provided their written informed consent to participate in the studies. The authors have authorized the display of their image in Figure 8.

## Author Contributions

JF and DM conceived, authored, and edited the manuscript. Both the authors contributed to the article and approved the submitted version.

### Conflict of Interest

The RespirAct™ is produced by Thornhill Medical, a for-profit spin-off from the University Health Network, to enable bone fide investigators to perform REB-sanctioned research. JF and DM have contributed to the development of the technology, and have shares in the company. JF sits on the Board of directors of the company.

## Footnotes

^2^https://tspace.library.utoronto.ca/handle/1807/89685
